# Comparison of Digital OPG and CBCT in Assessment of Risk Factors Associated with Inferior Nerve Injury during Mandibular Third Molar Surgery

**DOI:** 10.3390/diagnostics11122282

**Published:** 2021-12-06

**Authors:** Rakhi Issrani, Namdeo Prabhu, Mohammed Sghaireen, Hasna Rasheed Alshubrmi, Amal Mohamed Alanazi, Zainab Ali Alkhalaf, Mohammed Odhayd Alnusayri, Fahad Muqbil Aljohani, Zafar A. Khan

**Affiliations:** 1 Department of Preventive Dentistry, College of Dentistry, Jouf University, Sakaka 72341, Saudi Arabia; 2 Department of Oral & Maxillofacial Surgery and Diagnostic Sciences, College of Dentistry, Jouf University, Sakaka 72341, Saudi Arabia; dr.namdeo.prabhu@jodent.org (N.P.); dr.zafar.khan@jodent.org (Z.A.K.); 3 Department of Prosthetic Dental Sciences, College of Dentistry, Jouf University, Sakaka 72341, Saudi Arabia; dr.mohammed.sghaireen@jodent.org (M.S.); dr.hasna.alshubrmi@jodent.org (H.R.A.); amal-6677@hotmail.com (A.M.A.); dr.zainab.alkhalaf@jodent.org (Z.A.A.); dr.moalnusayri@jodent.org (M.O.A.); 4 Aja Primary Health Care Centre, Ministry of Health, Hail 55471, Saudi Arabia; faljohani19@moh.gov.sa

**Keywords:** panoramic, extraction, impacted molar, radiographic, inferior alveolar nerve

## Abstract

Background: Pre-operative radiographic assessment of the anatomical relationship between the roots of the mandibular third molar and the inferior alveolar nerve (IAN) is a must to minimize the risk of IAN injury during surgery. Objectives: To compare the radiographic signs of digital orthopantomogram (OPG) and cone-beam computed tomography (CBCT). An additional objective was to assess the cortex status between the mandibular canal and third molar on CBCT images in relation to the demographic characteristics, region (right or left side), and angulation of mandibular molar. Methodology: In this retrospective study, a total of 350 impacted mandibular third molars with a close relationship between the inferior alveolar canal (IAC) and impacted mandibular third molars on digital OPG were further referred for CBCT imaging for assessment of the position of the mandibular canal. The study was conducted between August 2018 and February 2020. Digital OPGs were evaluated for radiographic signs like interruption of the mandibular canal wall, darkening of the roots, diversion of the mandibular canal, and narrowing of the mandibular canal. The age and sex of patients, site of impacted third molar, Winter’s classification of mandibular third molar, position of IAC relative to impacted molar, and the radiographic markers of OPG were assessed for cortical integrity using CBCT. Chi square testing was applied to study the values of difference and binomial logistic regression was done to assess the factors associated with cortication. Statistical significance was set at *p* ≤ 0.05. Results: Among 350 patients, 207 (59.1%) were male and 143 (40.9%) were female with a mean age of 36.8 years. The most common OPG sign was interruption of white line, seen in 179 (51.1%) cases. In total, 246 cases (70.3%) showed an absence of canal cortication between the mandibular canal and the impacted third molar on CBCT images. Cortication was observed in all cases with a combination of panoramic signs which was statistically significant (*p* = 0.047). Cortication was observed in 85 (50.6%) cases where IAC was positioned on the buccal side, 11 (16.9%) in cases of inferiorly positioned IAC, and just 8 (7.6%) for cases of lingually positioned IAC which was statistically significant (*p* = 0.003). Statistically insignificant (*p* > 0.05) results were noted for cortex status in CBCT images with regards to the age, sex, site, and angulation of impacted third molars. Conclusion: CBCT imaging is highly recommended for those cases where diversion of the mandibular canal is observed on OPG and when the roots are present between canals.

## 1. Introduction

Surgical extraction of an impacted mandibular third molar, either for therapeutic or prophylactic purposes, is a common procedure in oral surgery [1,2]. It is often linked with complications like pain, swelling, bleeding, dry socket, infection, and trismus, which are often temporary in nature [3,4]. The most troublesome complication of all is temporary or permanent damage to the inferior alveolar nerve (IAN) [3]. The known risk of transient IAN injury after mandibular third molar surgery ranges from 0.6–5.3%, whereas the risk of permanent IAN damage is <1% [3]. Not only do such complications force the patients to undergo the ordeal of sensation loss, but the medicolegal implications are also extremely significant [5,6].

A careful pre-operative radiographic examination before surgery is useful in predicting complications [2]. Commonly employed radiographic techniques for this purpose are periapical radiographs, orthopantomogram (OPG), cone-beam computed tomography (CBCT), DentaScan, and computed tomography (CT) [3]. OPG is often used for initial examination that aids in assessing the root morphology, angulation of impacted tooth, and type of impaction [7,8]. However, being a 2D radiographic modality, OPG cannot be used to assess the spatial relationship between the inferior alveolar canal (IAC) and the impacted lower third molar [9,10]. Moreover, these images can be overlapped or distorted, which may lead to misinterpretation of results or incorrect judgements by clinicians [11]. In order to overcome these drawbacks of OPG, the use of CBCT has recently increased [12]. CBCT improves pre-operative surgical risk assessment by assisting the oral surgeon to outline the safe areas and the danger zones to gauge the direction of luxation [10,11,13].

There are seven radiographic signs that are commonly used as markers of a close association between the IAC and the lower third molar tooth. Of these, four are seen in relation to the root of a tooth (bifid root apex, deflection, narrowing, and darkening of the root) whereas the other three are related to changes in the IAC appearance (narrowing, diversion, and interruption in the white line of the canal) [14,15,16]. These radiographic signs are regarded as the ‘standard markers’ for assessing the risk of IAN injury during extraction of the lower third molar [14,15,16]. Furthermore, 3D imaging studies have shown that the shape and position of the IAC, and the absence of cortication between the IAC and mandibular third molar, are three reliable radiological predictors of IAN injury [2,17].

There have been a number of studies done on this topic globally, but our local record lacks any research in this context. Therefore, this study aimed to assess and compare the radiographic signs of digital OPG (as both isolated findings and in association with each other) with the cortex status between the mandibular canal and lower third molar on CBCT images. An additional objective was to assess the cortex status between the mandibular canal and the mandibular third molar on CBCT images in relation to demographic characteristics, region of the lower molar (right or left), and angulation of mandibular molar.

## 2. Materials and Methods

### 2.1. Study and Sample Characteristics

An observational study was conducted in a hospital-based setting. Ethical approval was granted by the local bioethics committee (approval no. 5-01-43). This retrospective study involved image analysis of a dataset of patients who showed a close relationship between the IAC and impacted third molars on digital OPG and were referred for CBCT imaging for assessment of the position of the mandibular canal from August 2018 to February 2020.

### 2.2. Sample Size

Sample size was calculated based on the changes in the prevalence of impacted third molar in a recent study conducted by Qassadi et al. [18] in Saudi Arabia, in which 57.0% of the sample had a mandibular impacted third molar. Considering 95% CI and 80% power, 323 individuals were sufficient to detect a clinically significant difference of 10%. The total sample size was therefore fixed at 350, accounting for a 10% chance of drop outs and attrition.

N = (zα + zβ)P(1 − P)/d2

### 2.3. Inclusion and Exclusion Criteria

Inclusion criteria considered were (i) acceptable quality of OPG and CBCT scans; (ii) OPG showing any of the four radiological findings that are the predictors for IAN damage during third molar surgery; and (iii) only Saudi nationals. Exclusion criteria considered were (i) history of trauma/surgery to mandible; (ii) presence of pathology affecting the jaws; (iii) patients with uncontrolled systemic illnesses; (iv) incomplete medical history and clinical records of patients; and (v) mandibular third molar with an incomplete root formation.

**Figure 1:**
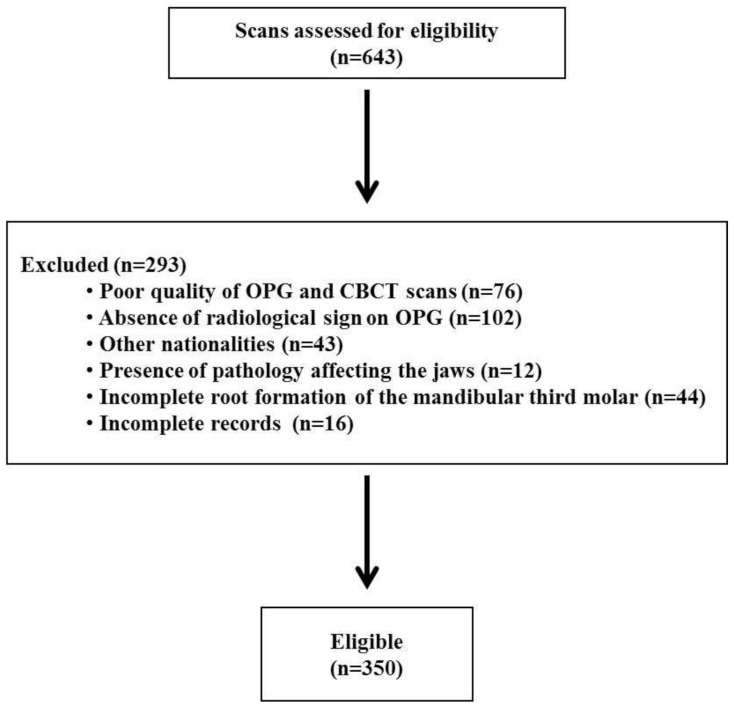
Flow chart of study participants.

Based on these criteria, a total of 350 scans were considered eligible for the study (Figure 1).

### 2.4. Evaluation of OPG Images

Digital OPG radiographs were acquired with an orthopantomography (Soredex Cranex D Digital Dental X-ray) unit, operating at 81 kVp/10 mA with an exposure time of 13.9 s and were reclaimed from hospital records. The images retrieved were those taken for the purpose of diagnosis and treatment planning of impacted mandibular third molars. The following radiographic signs that are considered to indicate a higher risk of IAN injury were evaluated on OPG [10,19]: (a) darkening of roots, (b) interruption of the white line of the canal, (c) diversion of the mandibular canal, and (d) narrowing of the mandibular canal. Occurrences of one or two radiographic signs were also evaluated.

Age and sex of the patients, region of molar (right or left), and angulation of mandibular molar in accordance with Winter’s classification [20] (mesio-angular, disto-angular, horizontal, or vertical) were also recorded. As per previous studies, age was converted to a categorical variable with a cut-off value of 30 years [12].

### 2.5. Analysis of CBCT Images

**Figure 2:**
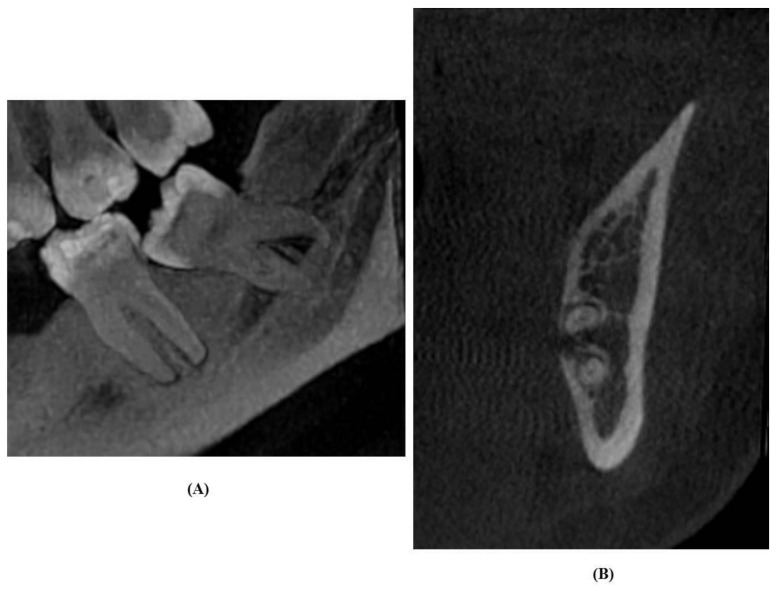
Panoramic images show darkening of the roots (**A**) and lingually positioned inferior alveolar canal with cortication on CBCT images (**B**).

**Figure 3:**
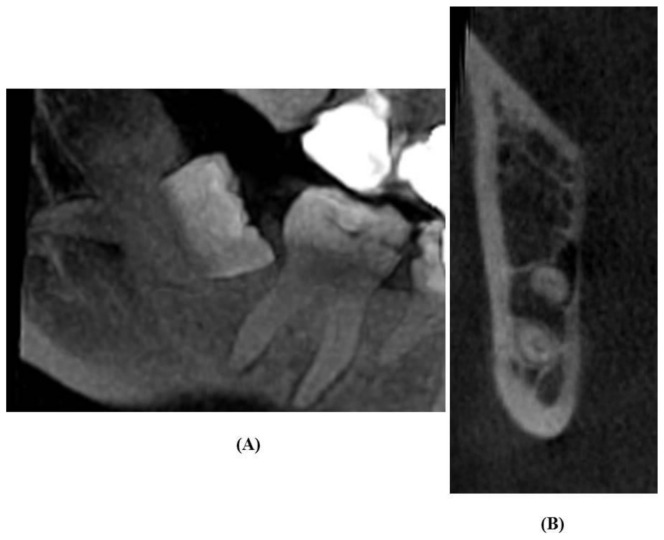
Panoramic images show darkening of the roots (**A**), and the inferior alveolar canal positioned between the roots without cortication on CBCT images (**B**).

**Figure 4:**
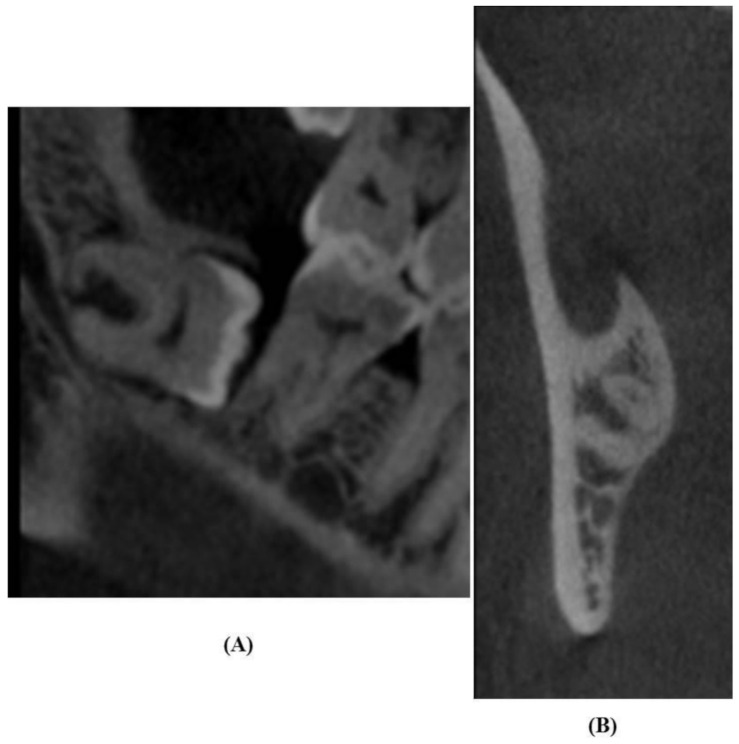
Panoramic images show diversion of mandibular canal (**A**), and buccally positioned inferior alveolar canal without cortication on CBCT images (**B**).

CBCT scans were reclaimed from the hospital records. Scans were acquired with a SCANORA 3Dx (Nahkelantie 160, Tuusula, Finland) set at 90 kV, 10 mA and scanning time of 20 s. On the CBCT images, the canal was traced and the image formed was seen in 3D view, i.e., sagittal, coronal, and axial planes under an extended field of view mode (100 × 100 mm) with standard resolution mode (voxel size of 0.25 mm). The acquired images were examined to determine the cortical layer integrity of the canal with respect to the root apex of the lower third molar (Figure 2, Figure 3 and Figure 4). Based on Ghaeminia classification [21], the position of the canal was also examined (buccal, lingual, between the roots, or inferiorly at the point of closest contact with the third molar root apex), along with the absence or presence of canal cortication. Data were evaluated using On Demand 3D software version 1.0.10.6388 (Yuseong-gu, Daejeon, Korea). The images were displayed on a TFT 27-inch monitor with 1280 × 1024 pixel screen resolution. The selected radiographs were independently examined by an oral radiologist and an oral and maxillofacial surgeon. All interpretations were done as per accepted standards, and any conflicts were decided by consensus.

### 2.6. Statistical Analysis

Comparison between various study parameters and CBCT findings was performed using chi square tests. Binomial logistic regression was done to assess the factors associated with cortication. Statistical analysis was performed with SPSS software version 21.0 (IBM Corp, Armonk, NY, USA). Statistical significance was set at *p* ≤ 0.05.

## 3. Results

Of the 350 CBCT images that were examined, cortication was present in 104 (29.7%) and absent in 246 (70.3%).

Table 1 depicts the distribution of presence of cortication with various demographic and clinical variables. Presence of cortication was observed in 31.8% of the participants who were in the ≤30 year age group and 28.4% in the above 30 year age group. Cortication in males was slightly more common than in their female counterparts (31.8 vs. 27.3) which was not statistically significant. Cortication on the left side (32%) was more common than on the right side (27.3%) of the mandible. Winter’s vertical impacted mandibular third molar was associated with greater cortication prevalence than other classifications, i.e., 32.2% followed by horizontal (32.2%), mesio-angular (26.6%), and disto-angular (17.6%), which was statistically not significant. Cortication was observed in 50.6% of the cases where IAC was positioned on the buccal side, 16.9% of cases with inferior positioned IAC, and just 7.6% of cases with lingual positioned IAC, which was statistically significant (*p* = 0.003).

Presence of cortication was compared with various signs observed in panoramic images, as shown in Table 2. Cortication was associated with 35.2% of cases with interruption of the canal wall, 30.4% with darkening of the root, 29.8% with narrowing of the canal, and 10.7% with diversion of the mandibular canal, and cortication was observed in all cases with a combination of panoramic signs that was statistically significant (*p* = 0.047).

Binomial logistic regression was done to assess the factors associated with cortication. Factors found to be significant in bivariate analysis were included in the regression model, i.e., position of IAC and radiographic findings were the only two factors included in the model. Position of IAC was found to be a predictor of cortication (odds = 2.1 and *p* = 0.02), as shown in Table 3.

## 4. Discussion

It is a well-established fact that surgical extraction of the third molars, especially those which are deep-seated below the cemento-enamel junction of the second molar, carries a high risk of IAN damage. Hence, a detailed examination of this anatomical relationship using radiographic techniques constitutes an inevitable pre-operative assessment tool [5].

Although OPG has been the mainstay radiographic technique for examining various risk factors related to third molars for many years, with the advancements of newer techniques, it has become imperative to study these newer techniques and also to compare them with the established techniques [2]. The prime objective of this study was to determine the usefulness of CBCT compared to OPG and whether it really helps us to understand the risk factors better. Additionally, taking repeated radiographs in cases of doubt leads to unnecessary radiation exposure for the patients. These doubts can be resolved by taking just one CBCT scan. This study aimed to examine the cortication status between the third molar and IAC in CBCT images in relation to the radiographic markers on OPG in the Saudi subpopulation.

Sedaghatfar et al. [22] showed that the OPG signs that are significantly related to IAN exposure following third molar extraction are the darkening of the root, interruption of the white line of the canal, diversion of the mandibular canal, and narrowing of the canal. These OPG signs were analyzed in this study, and the most frequent radiographic signs were interruption of the white line (51.1%) followed by darkening of the roots (19.7%). These findings are in accordance with other studies [10,15,19,23,24,25,26,27,28,29,30]. However, according to Ghai and Choudhury [8], Rood and Shehab [15], and Sedaghatfar et al. [22], darkening of roots was found to be the most common, while interruption of white line was the second most common OPG feature. Several studies have concluded that the presence of two or more signs on OPG indicates an increased risk of injury to IAN. [10,19,31,32,33,34,35]. In 4.9% of the cases studied in the current study, more than one radiographic sign was seen, which was fewer than in the study done by Pandey et al. [30] in which 19% of the cases had more than one sign. These different findings across previous studies might be attributed to sample variation, differences in investigators’ experience, use of different radiographic machines, and methodological diversity.

Recently, the cortication status between the mandibular third molar and IAC has been shown to be a reliable predictor of IAN exposure [2]. Previous studies based on CBCT and CT imaging techniques have shown increased paresthesia in cases with cortical disruption. [29,35,36,37,38,39,40]. In this study, 104 (29.7%) of the mandibular third molars showed cortication whereas 246 cases (70.3%) showed an absence of cortication. The percentage of absence of cortication in the present study was far greater than that noted by Kim et al. [7] Pandey et al. [30], and Waseem et al. [41], where in 61%, 63.8%, and 53.9% of CBCT images respectively, the IAN was actually in contact with the root of third molar. With respect to the OPG signs, diversion of canal showed the highest number of cases with absence of cortication between the third molar and IAC in CBCT images. This was similar to the study done by Tassoker [2] in which diversion of canal in OPG was found to be the only risk factor for the absence of cortication, with a roughly 12 times higher risk than other OPG signs. In another study, diversion of canal was also concluded to be the best diagnostic marker, followed by darkening of the root and interruption of the white line of the canal [21]. In this study, the other OPG signs associated with absence of cortication in CBCT images in sequence following the diversion of the canal were narrowing of the canal (70.2%) followed by darkening of the root (69.6%) and interruption of white line (64.8%). Fauzi et al. [35], Nakagawa et al. [40], Waseem et al. [41], and Kursun et al. [42] noted in their respective studies that interruption of the white line of the canal on OPG was significantly associated with a greater chance of having an IAC wall defect. Monaco et al. [26] and Dalili et al. [43] in their respective studies reported that they were more likely to find a defective IAC wall when there was narrowing of the root on OPG images. In 4.9% of the cases with two or more signs on OPG, absence of cortication in CBCT images was seen and this association was found to be statistically significant (*p* < 0.05). Pandey et al. [30] reported similar findings. Of all the images with two or more signs on OPG, darkening of roots in combination with interruption in the white line (2.3%) was noted in most of the cases. Similar findings were noted by Ghai and Choudhury, [8] Neves et al. [10], and Saha et al. [44].

This study revealed that in relation to the mandibular third molar, the majority of the IACs had a buccal course (48.0%) followed by lingual (30.0%), inferior (18.6%), and inter-radicular (3.4%) courses. The rare occurrence of inter-radicular course might be attributable to the fact that third molars with three or more roots or in a tilted position are rare [45]. The literature shows variable results in this regard. Few studies have reported the buccal position of the mandibular canal to be most common [19,45,46,47], whereas others have shown the inferior course as the most common course [2,8,11,48]. Meanwhile, Dalili et al. [43] and Pippi and Santoro [49] reported that most mandibular third molar roots are located on the lingual side of the canal. In our study, a statistically significant relationship was seen between the position of the IAC in relation to the mandibular third molar and cortication status (*p* < 0.05). Absence of cortication was noted most commonly for the inter-radicular course (100%), followed by lingual (92.4%), inferior (83.1%), and buccal courses (49.4%). Similar findings were noted in recent studies wherein the same two types (roots surrounding the canal and roots located on lingual side of the canal) were found to be most likely to have defects on the canal wall [2,13,46,50]. On the other hand, according to Tassoker [2], Ghai and Choudhury [8], and Nemsia et al. [51] lingually positioned IACs pose a high risk for IAN damage. Pandey et al. [30] and Xu et al. [52] also found in their respective studies that IAN injury is common if the third molar intersects with the mandibular canal, especially on the buccal side. The relationship between the mandibular canal and the root of the third molar is clinically important as it plays a significant role in the removal of buccal bone, tooth sectioning, and placement of elevator and direction of tooth removal [21,30].

In this study, impaction of the third molar was 59.1% in men and 40.9% in women. The sex difference regarding the impacted lower third molar varies from study to study. Previous studies have shown that women have a higher prevalence of impaction [24,27,53,54] whereas other studies have shown that men have a higher prevalence of impaction [7,55]. Nakagawa et al. [40] reported a significant difference in relation to mandibular canal defects between males and females; the defect rate for females was higher. However, no significant difference between males and females was found in our study. This study result was the same as those reported by Tassoker [2], Chen et al. [13] Nemsia et al. [52], and Cheung et al. [54].

In this study, based on Winter’s classification [20], mesio-angular was the most common type (54.9%) followed by vertical (23.4%), horizontal (16.8%), and disto-angular (4.9%). This was in agreement with Sedaghatfar et al. [22], Gomes et al. [23], Wassem et al. [41], Quek et al. [53], Deshpande et al. [56], and Nyugen et al. [57] In contrast, Kim et al. [7] and Tantanapornkul et al. [19] revealed that the horizontal angulation was the most common, followed by angular and vertical, whereas according to Chen et al. [13], Bataineh et al. [58], and Almendros-Marques et al. [59], vertical impaction was the most common type. In this study, canal defects were most frequently seen with disto-angular impaction (82.4%). Although vertical impaction was the second most common impaction type, it had a minimum risk for canal wall defects.

In this study, 37.7% of scans belonged to patients aged ≤30 years, whereas 62.3% of scans were of patients aged >30 years. The average age was 36.8 years. There was an insignificant correlation between age and cortication status. Using multivariate logistic analysis, Kubota et al. [12], Selvi et al. [60], and Korkmaz et al. [61] showed a significant association of increased age with IAN damage. In contrast, Nemsia et al. [51], Hasegawa et al. [62], and Shiratori et al. [63] noted an insignificant correlation between the proximity of the mandibular third molar to the canal and increasing age. Bigagnoli et al. [45], Deshpande et al. [56], and Nyugen et al. [57] documented that the risk of IAN injury increases with age, as observed in this study. The age-dependent increase in IAN injury risk may be associated with age-related factors like decreased bone elasticity, increased bone density, higher incidence of hypercementosis, narrowed periodontal space, and delayed regeneration process of the injured nerve due to decreased vascularization [12]. However, Chen et al. [13] suggested that patients between 18 and 30 years of age had the greatest chance of having a mandibular canal wall defect, while patients older than 60 years of age had a minimal risk. This difference might be because most patients have their mandibular third molars removed when they are young.

In this study, the incidence of impacted mandibular molars according to the site was almost equal, which was in contrast with the findings of Deshpande et al. [56] and Tay and Go [64] wherein impacted mandibular molars were more common on the right and left sides, respectively. This finding could be attributed to the variations in sample size involved. It was also noted in this study that the site (right/left) was not associated with cortication status, as also opined by Tassoker [2].

To summarize the findings of the current study, controversial results are reported in which a few researchers claim that advanced imaging techniques like CBCT have higher accuracy in the prediction of IAN exposure as compared to OPG [15,56], whereas others report no statistical difference between the two radiographic techniques [22,40]. According to the findings of the current study, CBCT is recommended when radiographic signs of diversion of the mandibular canal appear in OPG and when IAC position to the mandibular third molar is inter-radicular/lingual. However, in view of the socioeconomic conditions of some developing countries, the high cost of advanced radiographic techniques clearly justifies the more frequent usage of OPG as a pre-operative assessment tool in third molar surgery, although its predictive value is low with regards to the emergence of complications during or after the oral surgical procedure.

## 5. Limitations

The study had several limitations. First, it cannot be concluded that the study reflects the overall characteristics of the Saudi population, as it was short-term research conducted in a single institution. Second, the study only included patients who underwent OPG and CBCT, instead of using random sample collection; therefore, caution must be taken while interpreting the results.

## 6. Conclusions

In conclusion, the frequent absence of cortication as detected on CBCT images should raise red flags for surgeons while planning mandibular third molar surgery. The diversion of the canal as seen on OPG, which is statistically related to a higher risk of absence of cortication, should be considered a predictor of IAN damage. When this specific sign is observed, use of 3D imaging techniques is highly recommended. Additionally, the association of an inter-radicular/lingual course of the IAC with the absence of cortication also requires extra care by the surgeon while performing the surgical extraction of mandibular third molar. Finally, the data and results of this study can be used to set the direction of future research.

## Figures and Tables

**Table 1:** Comparison between prevalence of cortication and various study parameters.

Variables		Presence of Cortication n (%)	Absence of Cortication n (%)	Total Number of Cases n (%)	*p* Value
Age	≤30 years	42 (31.8)	90 (68.2)	132 (37.7)	0.503
	>30 years	62 (28.4)	156 (71.6)	218 (62.3)	
Sex	Male	65 (31.4)	142 (68.6)	207 (59.1)	0.553
	Female	39 (27.3)	104 (72.7)	143 (40.9)	
Site of mandibular third molar	Left	57 (32%)	121 (68%)	178 (50.9)	0.429
	Right	47 (27.3%)	125 (72.7%)	172 (49.1)	
Winter’s classification for angulation of impacted mandibular third molar	Mesio-angular	51 (26.6)	141 (73.4)	192 (54.9)	0.141
	Disto-angular	3 (17.6)	14 (82.4)	17 (4.9)	
	Horizontal	19 (32.2)	40 (67.8)	59 (16.8)	
	Vertical	31 (37.8)	51 (62.2)	82 (23.4)	
Position of IAC in relation to the mandibular third molar	Buccal side	85 (50.6)	83 (49.4)	168 (48.0)	0.003
	Lingual side	8 (7.6)	97 (92.4)	105 (30.0)	
	Inferior	11 (16.9)	54 (83.1)	65 (18.6)	
	Inter-radicular	00 (0.0)	12 (100)	12 (3.4)	

IAC: inferior alveolar canal.

**Table 2:** Relationships between signs on panoramic images and cortication status.

Signs on Panoramic Images	Presence of Cortication n (%)	Absence of Cortication n (%)	Total Number of Cases n (%)	*p* Value
Interruption of white line	63 (35.2)	116 (64.8)	179 (51.1)	0.047
Darkening of roots	21 (30.4)	48 (69.6)	69 (19.7)	
Diversion of mandibular canal	3 (10.7)	25 (89.3)	28 (8.0)	
Narrowing of mandibular canal	17 (29.8)	40 (70.2)	57 (16.3)	
DR + DMC	0 (0.0)	3(100)	3 (0.9)	
DR + NMC	0 (0.0)	1(100)	1 (0.3)	
DR + IWL	0 (0.0)	8(100)	8 (2.3)	
DMC + NMC	0 (0.0)	0 (0.0)	0 (0.0)	
DMC + IWL	0 (0.0)	1(100)	1 (0.3)	
NMC + IWL	0 (0.0)	4(100)	4 (1.1)	
Total	104 (29.7)	246 (70.3)	350 (100)	

IWL: interruption of white line; DR: darkening of roots; DMC: diversion of mandibular canal; NMC: narrowing of mandibular canal.

**Table 3:** Binomial logistic regression to predict the factors associated with cortication.

Variables	B	S.E.	Wald	df	Sig.	Exp (B)	95% C.I. for EXP(B)	
							Lower	Upper
Interruption of white line			5.298	8	0.725			
Darkening of roots	−18.515	19,898.166	0.000	1	0.999	0.000	0.000	
Diversion of mandibular canal	−19.044	19,898.166	0.000	1	0.999	0.000	0.000	
Narrowing of mandibular canal	−17.836	19,898.166	0.000	1	0.999	0.000	0.000	
DR + DMC	−19.787	19,898.166	0.000	1	0.999	0.000	0.000	
DR + NMC	0.545	30,568.425	0.000	1	1.000	1.725	0.000	
DR + IWL	0.545	44,848.767	0.000	1	1.000	1.725	0.000	
DMC + IWL	−0.242	24,451.408	0.000	1	1.000	0.785	0.000	
NMC + IWL	−0.242	44,848.767	0.000	1	1.000	0.785	0.000	
Position of IAC	3.787	5.682	2.334	1	0.0248	2.197	0.578	8.356
Constant	18.296	19,898.166	0.000	1	0.999	88,284,625.327		

## Data Availability

The data set used in the current study will be made available on request from Rakhi Issrani; riissrani@ju.edu.sa.

## References

[1] Mukherjee S., Vikraman B., Sankar D., Veerabahu M.S. (2016). Evaluation of outcome following coronectomy for the management of mandibular third molars in close proximity to inferior alveolar nerve. J. Clin. Diagn. Res..

[2] Di Nardo D., Mazzucchi G., Lollobrigida M., Passariello C., Guarnieri R., Galli M., De Biase A., Testarelli L. (2019). Immediate or delayed retrieval of the displaced third molar: A review. J. Clin. Exp. Dent..

[3] Tassoker M. (2019). Diversion of the mandibular canal: Is it the best predictor of inferior alveolar nerve damage during mandibular third molar surgery on panoramic radiographs?. Imaging Sci. Dent..

[4] Liu W., Yin W., Zhang R., Li J., Zheng Y. (2015). Diagnostic value of panoramic radiography in predicting inferior alveolar nerve injury after mandibular third molar extraction: A meta-analysis. Aust. Dent. J..

[5] Pathak S., Mishra N., Rastogi M.K., Sharma S. (2014). Significance of radiological variables studied on orthopantamogram to predict post-operative inferior alveolar nerve paresthesia after third molar extraction. J. Clin. Diagn. Res..

[6] Elkhateeb S.M., Awad S.S. (2018). Accuracy of panoramic radiographic predictor signs in the assessment of proximity of impacted third molars with the mandibular canal. J. Taibah Univ. Med. Sci..

[7] Kim H.J., Jo Y.J., Choi J.S., Kim H.J., Kim J., Moon S.Y. (2021). Anatomical risk factors of inferior alveolar nerve injury association with surgical extraction of mandibular third molar in Korean population. Appl. Sci..

[8] Ghai S., Choudhury S. (2018). Role of panoramic imaging and cone beam ct for assessment of inferior alveolar nerve exposure and subsequent paresthesia following removal of impacted mandibular third molar. J. Maxillofac. Oral Surg..

[9] Sghaireen M.G., Srivastava K.C., Shrivastava D., Ganji K.K., Patil S.R., Abuonq A., Mousa M.A., Dar-Odeh N., Sghaireen G.M., Kamal M.A. (2020). A CBCT based three-dimensional assessment of mandibular posterior region for evaluating the possibility of bypassing the inferior alveolar nerve while placing dental implants. Diagnostics.

[10] Neves F.S., Souza T.C., Almeida S.M., Haiter-Neto F., Freitas D.Q., Boscolo F.N. (2012). Correlation of panoramic radiography and cone beam CT findings in the assessment of the relationship between impacted mandibular third molars and the mandibular canal. Dentomaxillofac. Radiol..

[11] Zain-Alabdeen E.H., Alhazmi R.A., Alsaedi R.N., Alouf A.A., Alahmady O.A. (2020). Preoperative cone beam computed tomography evaluation of mandibular second and third molars in relation to the inferior alveolar canal. Saudi J. Health Sci..

[12] Kubota S., Imai T., Nakazawa M., Uzawa N. (2020). Risk stratification against inferior alveolar nerve injury after lower third molar extraction by scoring on cone-beam computed tomography image. Odontology.

[13] Chen Y., Liu J., Pei J., Liu Y., Pan J. (2018). The risk factors that can increase possibility of mandibular canal wall damage in adult: A cone-beam computed tomography (CBCT) study in a Chinese population. Med. Sci. Monit..

[14] Su N., van Wijk A., Berkhout E., Sanderink G., De Lange J., Wang H., van der Heijden G.J. (2017). Predictive value of panoramic radiography for injury of inferior alveolar nerve after mandibular third molar surgery. J. Oral Maxillofac. Surg..

[15] Rood J.P., Shehab B.A. (1990). The radiological prediction of inferior alveolar nerve injury during third molar surgery. Br. J. Oral Maxillofac. Surg..

[16] Palma-Carrio C., Garcia-Mira B., Larrazabal-Moron C., Penarrocha-Diago M. (2010). Radiographic signs associated with inferior alveolar nerve damage following lower third molar extraction. Med. Oral Patol. Oral Cir. Bucal..

[17] Patel P.S., Shah J.S., Dudhia B.B., Butala P.B., Jani Y.V., Macwan R.S. (2020). Comparison of panoramic radiograph and cone beam computed tomography findings for impacted mandibular third molar root and inferior alveolar nerve canal relation. Indian J. Dent. Res..

[18] Qassadi T.M., Shafei A.A., Alhazmi A.A., Odabi N.I. (2020). Prevalence and pattern of third molar impaction among the Saudi Population in Jazan Region, Saudi Arabia. Saudi J. Oral Dent. Res..

[19] Tantanapornkul W., Okouchi K., Fujiwara Y., Yamashiro M., Maruoka Y., Ohbayashi N., Kurabayashi T. (2007). A comparative study of cone-beam computed tomography and conventional panoramic radiography in assessing the topographic relationship between the mandibular canal and impacted third molars. Oral Surg. Oral Med. Oral Pathol. Oral Radiol. Endod..

[20] Winter G.B. (1926). Impacted Mandibular Third Molar.

[21] Ghaeminia H., Meijer G.J., Soehardi A., Borstlap W.A., Mulder J., Berge S.J. (2009). Position of the impacted third molar in relation to the mandibular canal. Diagnostic accuracy of cone beam computed tomography compared with panoramic radiography. Int. J. Oral Maxillofac. Surg..

[22] Sedaghatfar M., August M.A., Dodson T.B. (2005). Panoramic radiographic findings as predictors of inferior alveolar nerve exposure following third molar extraction. J. Oral Maxillofac. Surg..

[23] Gomes A.C., Vasconcelos B.C., Silva E.D., Ade F.C., Neto I.C.P. (2008). Sensitivity and specificity of pantomography to predict inferior alveolar nerve damage during extraction of impacted lower third molars. J. Oral Maxillofac. Surg..

[24] Szalma J., Lempel E., Jeges S., Szabó G., Olasz L. (2010). The prognostic value of panoramic radiography of inferior alveolar nerve damage after mandibular third molar removal: Retrospective study of 400 cases. Oral Surg. Oral Med. Oral Pathol. Oral Radiol. Endod..

[25] Khan I., Halli R., Gadre P., Gadre K.S. (2011). Correlation of panoramic radiographs and spiral CT scan in the preoperative assessment of intimacy of the inferior alveolar canal to impacted mandibular third molars. J. Craniofac. Surg..

[26] Monaco G., Montevecchi M., Bonetti G.A., Gatto M.R., Checchi L. (2004). Reliability of panoramic radiography in evaluating the topographic relationship between the mandibular canal and impacted third molars. J. Am. Dent. Assoc..

[27] Jerjes W., El-Maaytah M., Swinson B., Upile T., Thompson G., Gittelmon S., Baldwin D., Hadi H., Vourvachis M., Abizadeh N. (2006). Inferior alveolar nerve injury and surgical difficulty prediction in third molar surgery: The role of dental panoramic tomography. J. Clin. Dent..

[28] Hasani A., Ahmadi Moshtaghin F., Roohi P., Rakhshan V. (2017). Diagnostic value of cone beam computed tomography and panoramic radiography in predicting mandibular nerve exposure during third molar surgery. Int. J. Oral Maxillofac. Surg..

[29] Blaeser B.F., August M.A., Donoff R.B., Kaban L.B., Dodson T.B. (2003). Panoramic radiographic risk factors for inferior alveolar nerve injury after third molar extraction. J. Oral Maxillofac. Surg..

[30] Pandey R., Ravindran C., Pandiyan D., Gupta A., Aggarwal A., Aryasri S. (2018). Assessment of Roods and Shehab criteria if one or more radiological signs are present in orthopantomogram and position of the mandibular canal in relation to the third molar apices using cone beam computed tomography: A radiographic study. Tanta Dent. J..

[31] Jhamb A., Dolas R.S., Pandilwar P.K., Mohanty S. (2009). Comparative efficacy of spiral computed tomography and orthopantomography in preoperative detection of relation of inferior alveolar neurovascular bundle to the impacted mandibular third molar. J. Oral Maxillofac. Surg..

[32] Szalma J., Lempel E., Jeges S., Olasz L. (2011). Darkening of third molar roots: Panoramic radiographic associations with inferior alveolar nerve exposure. J. Oral Maxillofac. Surg..

[33] Bell G.W. (2004). Use of dental panoramic tomographs to predict the relation between mandibular third molar teeth and the inferior alveolar nerve. Radiological and surgical findings, and clinical outcome. Br. J. Oral Maxillofac. Surg..

[34] Nakamori K., Fujiwara K., Miyazaki A., Tomihara K., Tsuji M., Nakai M., Michifuri Y., Suzuki R., Komai K., Shimanishi M. (2008). Clinical assessment of the relationship between the third molar and the inferior alveolar canal using panoramic images and computed tomography. J. Oral Maxillofac. Surg..

[35] Fauzi A.A., Nazimi A.J., Rashdi M.F., Fouzi N., Kamarudin N.A., Ramli R. (2019). Interruption regions in the white line: A novel panoramic finding in the risk assessment of mandibular canal exposure by third molar. J. Clin. Diagn. Res..

[36] Ueda M., Nakamori K., Shiratori K., Igarashi T., Sasaki T., Anbo N., Kaneko T., Suzuki N., Dehari H., Sonoda T. (2012). Clinical significance of computed tomographic assessment and anatomic features of the inferior alveolar canal as risk factors for injury of the inferior alveolar nerve at third molar surgery. J. Oral Maxillofac. Surg..

[37] Susarla S.M., Sidhu H.K., Avery L.L., Dodson T.B. (2010). Does computed tomographic assessment of inferior alveolar canal cortical integrity predict nerve exposure during third molar surgery?. J. Oral Maxillofac. Surg..

[38] Nakamori K., Tomihara K., Noguchi M. (2014). Clinical significance of computed tomography assessment for third molar surgery. World. J. Radiol..

[39] Park W., Choi J.W., Kim J.Y., Kim B.C., Kim H.J., Lee S.H. (2010). Cortical integrity of the inferior alveolar canal as a predictor of paresthesia after third-molar extraction. J. Am. Dent. Assoc..

[40] Nakayama K., Nonoyama M., Takaki Y., Kagawa T., Yuasa K., Izumi K., Ozeki S., Ikebe T. (2009). Assessment of the relationship between impacted mandibular third molars and inferior alveolar nerve with dental 3-dimensional computed tomography. J. Oral Maxillofac. Surg..

[41] Waseem N., Asim M.A., Maqsood A., Ghafoor M.W., Mirza N., Khalid M.O. (2021). Evaluation of patterns of impacted third molars and their association with vital structures by radiographic examination. Pak. Armed Forces Med. J..

[42] Kursun S., Hakan K.M., Bengi O., Nihat A. (2015). Use of cone beam computed tomography to determine the accuracy of panoramic radiological markers: A pilot study. J. Dent. Sci..

[43] Dalili Z., Mahjoub P., Sigaroudi A.K. (2011). Comparison between cone beam computed tomography and panoramic radiography in the assessment of the relationship between the mandibular canal and impacted class C mandibular third molars. Dent. Res. J. (Isfahan).

[44] Saha N., Kedarnath N.S., Singh M. (2019). Orthopantomography and cone-beam computed tomography for the relation of inferior alveolar nerve to the impacted mandibular third molars. Ann. Maxillofac. Surg..

[45] Bigagnoli S., Greco C., Costantinides F., Porrelli D., Bevilacqua L., Maglione M. (2021). CBCT radiological features as predictors of nerve injuries in third molar extractions: Multicenter prospective study on a northeastern Italian population. Dent. J..

[46] Maegawa H., Sano K., Kitagawa Y., Ogasawara T., Miyauchi K., Sekine J., Inokuchi T. (2003). Pre-operative assessment of the relationship between the mandibular third molar and the mandibular canal by axial computed tomography with coronal and sagittal reconstruction. Oral Surg. Oral Med. Oral Pathol. Oral Radiol. Endod..

[47] Wang W.Q., Chen M.Y., Huang H.L., Fuh L.J., Tsai M.T., Hsu J.T. (2015). New quantitative classification of the anatomical relationship between impacted third molars and the inferior alveolar nerve. BMC Med. Imaging.

[48] Yabroudi F., Pedersen S.S. (2012). Cone beam tomography (CBCT) as a diagnostic tool to assess the relationship between the inferior alveolar nerve and roots of mandibular wisdom teeth. Smile Dent. J..

[49] Pippi R., Santoro M. (2015). A multivariate statistical analysis on variables affecting inferior alveolar nerve damage during third molar surgery. Br. Dent. J..

[50] Salam S., Rehman A.Z.U., Alam S., Jamil Y., Irshad M. (2020). Relative position of mandibular third molar and inferior alveolar nerve using cone beam computed tomography. Isra Med. J..

[51] Nemsia H., Tellili N., Bouanene I., Tlili M., Khenfir F., Khalfi M.S., ben Amor F. (2017). Classification of impacted mandibular third molars using cone beam computed tomography based on neurological risks: NRC. Med. Buccale Chir. Buccale.

[52] Xu G.Z., Yang C., Fan X.D., Yu C.Q., Cai X.Y., Wang Y., He D. (2013). Anatomic relationship between impacted third mandibular molar and the mandibular canal as the risk factor of inferior alveolar nerve injury. Br. J. Oral Maxillofac. Surg..

[53] Quek S.L., Tay C.K., Tay K.H., Toh S.L., Lim K.C. (2003). Pattern of third molar impaction in a Singapore Chinese population: A retrospective radiographic survey. Int. J. Oral Maxillofac. Surg..

[54] Cheung L.K., Leung Y.Y., Chow L.K., Wong M.C., Chan E.K., Fok Y.H. (2010). Incidence of neurosensory deficits and recovery after lower third molar surgery: A prospective clinical study of 4338 cases. Int. J. Oral Maxillofac. Surg..

[55] Bozzatello J. (2006). Relationship between craniofacial architecture and retained lower third molar. Its’ symptomatology. Rev. Fac. Cienc. Med. (Cordoba Argent.).

[56] Deshpande P., VGuledgud M., Patil K. (2013). Proximity of impacted mandibular third molars to the inferior alveolar canal and its radiographic predictors: A panoramic radiographic study. J. Maxillofac. Oral Surg..

[57] Nguyen E., Grubor D., Chandu A. (2014). Risk factors for permanent injury of inferior alveolar and lingual nerves during third molar surgery. J. Oral Maxillofac. Surg..

[58] Bataineh A.B., Albashaireh Z.S., Hazza’a A.M. (2002). The surgical removal of mandibular third molars: A study in decision making. Quintessence Int..

[59] Almendros-Marques N., Berini-Aytes L., Gay-Escoda C. (2006). Influence of lower third molar position on the incidence of preoperative complications. Oral Surg. Oral Med. Oral Pathol. Oral Radiol. Endod..

[60] Selvi F., Dodson T.B., Nattestad A., Robertson K., Tolstunov L. (2013). Factors that are associated with injury to the inferior alveolar nerve in high-risk patients after removal of third molars. Br. J. Oral Maxillofac. Surg..

[61] Korkmaz Y.T., Kayıpmaz S., Senel F.C., Atasoy K.T., Gumrukcu Z. (2017). Does additional cone beam computed tomography decrease the risk of inferior alveolar nerve injury in high-risk cases undergoing third molar surgery? Does CBCT decrease the risk of IAN injury?. Int. J. Oral Maxillofac. Surg..

[62] Hasegawa T., Ri S., Shigeta T., Akashi M., Imai Y., Kakei Y., Shibuya Y., Komori T. (2013). Risk factors associated with inferior alveolar nerve injury after extraction of the mandibular third molar—A comparative study of pre-operative images by panoramic radiography and computed tomography. Int. J. Oral Maxillofac. Surg..

[63] Shiratori K., Nakamori K., Ueda M., Sonoda T., Dehari H. (2013). Assessment of the shape of the inferior alveolar canal as a marker for increased risk of injury to the inferior alveolar nerve at third molar surgery: A prospective study. J. Oral Maxillofac. Surg..

[64] Tay A.B., Go W.S. (2004). Effect of exposed inferior alveolar neurovascular bundle during surgical removal of impacted lower third molars. J. Oral Maxillofac. Surg..

